# End-of-life decision-making in the neonatal intensive care unit

**DOI:** 10.3389/fped.2023.1352485

**Published:** 2024-01-08

**Authors:** Ana Morillo Palomo, Montse Esquerda Aresté, Ana Riverola de Veciana, Francisco José Cambra Lasaosa

**Affiliations:** ^1^ Neonatal Intensive Care Unit, Sant Joan de Déu Hospital, Barcelona, Spain; ^2^ Institut Borja de Bioètica, Universitat Ramon Llull, Barcelona, Spain; ^3^ School of Medicine, University of Lleida, Lleida, Spain; ^4^ Pediatric Intensive Care Unit, Sant Joan de Déu Hospital, Barcelona, Spain

**Keywords:** end-of-life, decision making, family-centred care, bioethics, neonatal care

## Abstract

Most paediatric deaths occur in the neonatal period, many of them in neonatal intensive care units after withdrawal of life support or the decision not to initiate new treatments. In these circumstances, discussions with families and decision-making are fundamental elements of the care and attention given to newborn babies. In this context, bioethical deliberation can help us to identify the values at stake, the different courses of action to be taken, and the means to ensure that family-shared decision-making is appropriate to the patient's situation and in accordance with the family's values.

## 1 Introduction

In the paediatric population, most children who die do so around the time of birth, or within the first month of life. A third of the deaths in the paediatric age group (0–18 years of age) occur in the neonatal period. Most of these deaths occur in the neonatal intensive care unit, many of them after withdrawal or non-initiation of life support—that is, after a decision has been made to move from the intention to reverse the clinical problem to palliative care (1, 2).

Advances in foetal and neonatal medicine have led to improvements in the accuracy of diagnosis and treatment of life-limiting conditions, with technology making it possible to increase the survival rate of patients who would have died a few years ago (3). However, in some of these cases, the prolongation of life through various technological support therapies can increase the suffering of both the babies and their families, in part by creating unrealistic expectations and hopes.

Prematurity at the threshold of viability, life-limiting congenital anomalies, severe and irreversible neurological lesions, multiple organ failure, irreparable lesion of a vital organ, and severe comorbidities are among the circumstances that neonatal units must deal with. Neonatologists are confronted with neonates in whom they are unsure whether to iniciate resuscitation, whether a new treatment is indicated, or whether alredy started support measures should be withdraw (4). Consequently, it is not always appropriate to do everything that is technically possible, and in some circumstances treatment with therapeutic intent must be replaced by palliative care aimed at relieving or preventing the suffering for these newborns and their families (5). Redirecting care to comfort measures at the appropriate time is essential to avoid futility and to allow these children to live and die with dignity (6).

Given this background, there is a need, sometimes an urgent need, to talk to families in order to reach consensus and make shared decisions about the care of these newborns.

## 2 Most relevant ethical considerations in decision-making in neonatology

The bioethical principles proposed by Beauchamp and Childress (beneficence, non- maleficence, justice, and autonomy) form the basis of bioethical reflection also in neonatology (7). However, the beginning of life gives rise to difficult scenarios which, when analysed from a principled point of view, result in a somewhat reductionist view of a problem that is in fact much broader. Thus, although the best interest of the child has been considered the core or fundamental bioethical principle for decision-making in paediatrics, there are various authors who disagree with this “axiom” or “precept” and propose a redefinition of the best interest of the child through the different approaches that have emerged throughout the history of neonatology (8, 9). The detractors of the best interest of the child as a central principle in the analysis of bioethical problems at the beginning of life point out that it is individualistic, it takes into account the interest of only one person, imprecise and difficult to determine, since the best interest of the child is determined by the parents and the physicians, and that, since it is based mainly on the quality of life, it can lead to approaches that are too life-centred, or, at the other extreme, to the renunciation of treatment that is in fact both appropriate and proportionate.

Another aspect of the best interest of the child in neonatology is that related to the principle of autonomy. From a legal and moral point of view, the parents are the ones who represent the autonomy of their child, an autonomy that is subrogated to them, and therefore, *a priori*, they are the ones who can best represent the best interest of their child (10, 11). It follows that parental decisions not only affect the child, but also have consequences for the entire family unit. In any case, the Principle of Autonomy in parental decisions does not have an absolute ethical value; in some cases, the best interest of the child may conflict with this parental right (12).

Against this background, some ethicists argue for a greater role for the principle of non-maleficence, as most appropriate in situations where parents and professionals may take opposing positions in decision making. Gillam introduced the concept of “the zone of parental discretion” as a tool for ethical deliberation in situations of disagreement, whether parents reject treatment proposed by professionals or request treatment that is not initially recommended (13). The zone of parental discretion can be defined as a protected ethical space in which parents can make legitimate decisions for their children, even if these decisions are not be the absolute best ones for their children, but at the same time are not so bad as to cause harm.

This concept opens up a much wider range of course of action than the principle of best interest, and offers a practical way of guiding decisions when parents and professionals disagree, because it focuses on what is potentially most important—not causing harm—rather than maximising the best interests of the child (13, 14). In this zone of parental discretion, Engelhardt's permission principle comes into play, which would facilitate decision-making between moral strangers (15). The principle of permission allows coexistence between people and communities that do not share the same moral content. The zone of parental discretion also allows the representation of the child's autonomy to be extended to the whole family—parents and siblings—facilitating the exercise of parental and family roles that are often interrupted or broken by the child's admission to the neonatal unit.

This zone of parental discretion has been fostered by the shift from a medicine focused on doctors' decisions, designed to protect the patient's interests from a paternalistic point of view, to a medicine in which the patient's autonomy prevails, perhaps to an excessive degree. This has led to a shift in the paradigm of the doctor-patient-family relationship, changing part of the social contract that corresponded to the physician, and thereby modifying, to a certain extent, the basis of medical professionalism (16).

This evolution and modification of roles in the professional-patient relationship pushes us forward by changing the balance of power in terms of aspects such as the values and beliefs of the different subjects involved in decision-making (17). Broadening the focus beyond the criterion of the best interest of the child and parental autonomy leads us to the concept of relational autonomy. Relational autonomy is based on the shared conviction that human beings are socially integrated and that the agents’ identities take shape in the context of their social relations, and are shaped by a complex web of interlocking social determinants (18). Relational autonomy emphasises social context and relationships and recognizes the inherent emotional aspects of decision-makers.

## 3 Characteristics of decision-making in neonatology

End-of-life decision-making is one of the practices in neonatology in which ethics plays a very important role. Professionals are often faced with difficult decisions with a high degree of clinical and ethical uncertainty (19). Clinical deliberation involving the conflicting values is the way to go in these complex scenarios. Deliberation allows for the evaluation of different options by examining the pros and cons of each course of action and choosing the one that seems most appropriate. Deliberation helps professionals work towards becoming more open, self-critical, and analytical, with the aim of reaching a definitive decision (4). Some authors are critical of the need for consensus among professionals, pointing out that when consensus is absolute, there is a risk of imposing professionals' values on the family. These authors stress that the existence of a certain degree of dissent among the professionals can help to suggest possible or intermediate courses of action to which the family may be more receptive (20).

Decision-making is an iterative process with a number of steps required to reach the desired goal. The process of decision making and the outcome of the decision are equally important (21). However, in end-of-life situations the ethical decision is often not a completely free choice. It may carry an undeniable emotional charge and may not be entirely in line with the values of the parents, who may sometimes seek absolute proof in order to eliminate uncertainty (22).

In neonatology, patient-centred goes hand in hand with family-centred care. In line with the philosophy of developmental and family-centred care, the best way forward is a shared approach to decision making, with varying degrees of involvement of the parents according to their capacities, needs, wishes, and values (23, 24). Habermas' dialogical or communicative ethics finds its paradigm in neonatal units that apply family-centred care (25).

Neonatal professionals must allow for and promote a space in which parents can feel comfortable to make their choices, with full recognition that perspectives may change throughout the decision-making process, in accordance with the evolution of the patient.

A climate of trust must be established and communication must be based on respect, with an attitude of care, empathy, and honesty. When there is disagreement between professionals and families it is crucial to find time and spaces to meet, as often as necessary, to maintain the dialogue about the situation and to hear each other's points of view in order to reach consensus.

We can identify three zones in this decision-making process: one that is beneficial to the patient and the family, one that is futile, and one that is a grey area. The grey zone is where we find uncertainty, unpredictability, and the urgent need to explore values. Decision-making is a process in which it is difficult to separate the emotional from the rational. Understanding these emotions can help us to clarify the values and personal priorities of the family (26).

## 4 Final reflections

Shared decision-making is a process that leads to a reconciliation of the technical dimension with the values involved in the decision. The aim is to align the values of the parents with the values associated with the treatment proposed by healthcare professionals. In this way, shared decision-making aims to ensure that the patients and/or their representatives understand the options available, with their pros and cons, and to ensure that patients' goals and preferences are used to guide the care decisions (27).

There is scarce information in the scientific literature about the nature of decision-making in neonatology. Most of the paediatric literature on shared decision-making does not discuss the neonatal period and are limited to the paediatric age. The majority of studies on the neonatal period focus on discussions and decisions at the limits of viability and extreme prematurity. Apart from these points, there are few references to decision-making in the framework of the neonatal intensive care unit and at the end of life. The bibliography is mainly concerned with the causes of death in the NICU, the characteristics of the death, and bereavement support for parents and health care professionals.

Shared decision-making has only been studied in recent years, mainly in the form of reviews and qualitative studies based on interviews with families and/or professionals (28, 29). In Switzerland, a study group was set up in 2012 to investigate the needs of paediatric patients at the end-of-life. Called PELICAN (Paediatric End of Life CAre Needs), it used a questionnaire to explore these needs and to assess parents' perspectives on the end of life of their neonatal and paediatric children. This is the only study found that has quantitatively assessed families' experiences at the end of their child's life (30).

Janvier is one of the authors who has done the most research on the communication with the families in the neonatal period, focusing on the appropriateness of treatment. She proposes the use of the mnemonic acronym SOBPIE (Situation, Opinions and Options, Basic interactions, Parents, Information, and Emotions) as an aid to having fruitful conversations with parents and taking full advantage of the conversations that develop (31).

Other authors, such as Lantos, have also looked at shared decision making, focusing on the problems that arise in making decision. This author argues that there is no such thing as a good decision or a bad decision, but that parents must understand that the decision made must be the best one for their child. He examines the degree of involvement that each of the parties involved must have in the decision-making process (32, 33).

Most authors advocate a humane, compassionate approach to the families, with honesty and empathy helping to create an appropriate atmosphere for shared decision making (34, 35). In addition, parents' perception that they have been involved in the decision making about their child's illness and death seems to help them to cope better with the process of grieving for their deceased child (36).

Shared decision-making between parents and professionals seems to be the way to achieve the greatest consensus on the interest of the newborn.

## References

[1] Ramelet AS Bergstraesser E Grandjean C Dorsaz A Fahrni-Nater P Cignacco E Comparison of end-of-life care practices between children with complex chronic conditions and neonates dying in ICU versus non-ICUs: a substudy of the pediatric end of life care needs in Switzerland (PELICAN) project. Pediatr Crit Care Med. (2020) 21(5):e236–46. 10.1097/PCC.0000000000002259 32091504

[2] Boize P Borrhormee S Michel P Betremieux P Hubert P Moriette G. Neonatal end-of-life decision-making almost 20 years after the EURONIC study: a French survey. Arch Pediatr. (2019) 26(6):330–6. 10.1016/j.arcped.2019.06.007 31353145

[3] Tejedor Torres JC López de Heredia Goya J Herranz Rubia N Nicolás Jiménez P García Muñoz F Pérez Rodríguez J. Recommendations on decision making and end-of-life care in neonatology. An Pediatr (Barc). (2013) 78(1):190.e1–e14. 10.1016/j.anpedi.2012.07.012 23022201

[4] Arnáez J Tejedor JC Caserio S Montes MT Moral MT González de Dios J Bioethics at the end of life in neonatology: unresolved issues. An Pediatr (Barc). (2017) 87(6):356.e1–e12. 10.1016/j.anpedi.2017.03.014 28476218

[5] Quinn M Weiss AB Crist JD. Early for everyone. Reconceptualizing palliative care in the neonatal intensive care unit. Adv Neonatal Care. (2020) 20(2):109–17. 10.1097/ANC.0000000000000707 31990696

[6] Mills M Cortezzo D. Moral distress in the neonatal intensive care unit: what is it, why it happens, and how we can address it. Front Pediatr. (2020) 10(8):581. 10.3389/fped.2020.00581 PMC7511509 33014949

[7] Beauchamp TL Childress F. Principles of Biomedical Ethics. New York: Oxford University Press (2001).

[8] González-Melado FJ Di Pietro ML. The best interest of the child in neonatology: is the best interest of the child? Cuad Bioet. (2015) 26(87):201–22. 26378595

[9] Bester JC. The best interest standard and children: clarifying a concept and responding to its critics. J Med Ethics. (2019) 45(2):117–24. 10.1136/medethics-2018-105036 30242078

[10] Guimaraes H Rocha G Bellieni C Buonocore G. Rights of the newborn and end-of-life decisions. J Matern-Fetal Neonatal Med. (2012) 25(Suppl 1):76–8. 10.3109/14767058.2012.665240 22372731

[11] Buchanan A Brock D Daniels N Wikler D. From Chance to Choice. Chapter: Why not the Best? Cambridge, United Kingdom: Cambridge University Press (2000). p. 156.

[12] Willems D Verhagen E van Wijlick E. Infants’ best interest in end-of-life care for newborn. Pediatrics. (2014) 134(4):e1163–8. 10.1542/peds.2014-0780 25246628

[13] Gillam L. The zone of parental discretion: an ethical tool for dealing with disagreement between parents and doctors about medical treatment for a child. Clin Ethics. (2016) 11(1):1–8. 10.1177/1477750915622033

[14] Albersheim S. The extremely preterm infant: ethical considerations in life-and-death decision-making. Front Pediatr. (2020) 26(8):55. 10.3389/fped.2020.00055 PMC7054342 32175292

[15] Engelhardt HT Jr. The Foundations of Bioethics. New York: Oxford University Press (1996).

[16] Prentice TM Gillam L. Can the ethical best practice of shared decision-making lead to moral distress? Bioeth Inq. (2018) 15(2):259–68. 10.1007/s11673-018-9847-8 29541925

[17] Kodadek MP Feeg VD. Using vignettes to explore how parents approach end of life decision making for terminally ill infants. Pediatr Nurs. (2002) 28(4):333–43. 12226955

[18] Gómez-Vírseda C De Maeseneer Y Gastmans C. Relational autonomy in end-of-life care ethics: a contextualized approach to real-life complexities. BMC Med Ethics. (2020) 21(1):50. 10.1186/s12910-020-00495-1 32605569 PMC7325052

[19] Messner H Gentili L. Reconciling ethical and legal aspects in neonatal intensive care. J Matern-Fetal Neonatal Med. (2011) 24(S1):126–8. 10.3109/14767058.2011.607672 21888497

[20] Wilkinson D Truog R Savulescu J. In favor of medical dissensus: why we should agree to disagree about end-of-life decisions. Bioethics. (2016) 30(2):109–18. 10.1111/bioe.12162 25908398 PMC4864446

[21] Kyounghae K Heinze K Xu J Kurtz M Park H Foradorl M Theories of health care decision making at the end of life: a meta-ethnography. West J Nurs Res. (2018) 40(12):1861–84. 10.1177/0193945917723010 28816094 PMC6474239

[22] Van Manen MA. On ethical (in)decisions experienced by parents of infants in neonatal intensive care. Qual Health Res. (2014) 24(2):279–87. 10.1177/1049732313520081 24469694

[23] Gillam L Sullivan J. Ethics at the end of life: who should make decisions about treatment limitation for young children with life-threating of life-limiting conditions? J Paediatr Health. (2011) 47(9):594–8. 10.1111/j.1440-1754.2011.02177.x 21951439

[24] Kon AA. Life and death choices in neonatal care: applying shared decision-making focused on parental values. Am J Bioeth. (2011) 11(2):35–6. 10.1080/15265161.2010.540067 21337275

[25] Habermas J. The Theory of Communicative Action: Reason and the Rationalization of Society. Boston: Beacon Press (1984).

[26] Haward MF Gaucher N Payot A Robson K Janvier A. Personalized decision making: practical recommendations for antenatal counseling for fragile neonates. Clin Perinatol. (2017) 44(2):429–45. 10.1016/j.clp.2017.01.006 28477670

[27] Walter JK Hwang JH Fiks AG. Pragmatic strategies for shared decision-making. Pediatrics. (2018) 142(s3):s157–62. 10.1542/peds.2018-0516F 30385622

[28] Janvier A Farlow B Baardsnes J Pearce R Barrington K. Measuring and communicating meaningful outcomes in neonatology: a family perspective. Semin Perinatol. (2016) 40(8):571–7. 10.1053/j.semperi.2016.09.009 27793420

[29] de Vos M Bos A Plötz F van Heerde M Graaff B Tates K Talking with parents about end-of-life decisions for their children. Pediatrics. (2015) 135(2):e465–76. 10.1542/peds.2014-1903 25560442

[30] Zimmermann K Bergstraesser E Engberg S Ramelet A-S Marfut-Russemberger K Von der Weid N When parents face the death of their child: a nationwide cross-sectional survey of parenteral perspectives on their child’s end-of-life. BCM Palliat Care. (2016) 15:30. 10.1186/s12904-016-0098-3 PMC4784404 26956995

[31] Janvier A Barrington K Farlow B. Communication with parents concerning withholding or withdrawing of life-sustaining interventions in neonatology. Semin Perinatol. (2014) 38(1):38–46. 10.1053/j.semperi.2013.07.007 24468568

[32] Blumenthal-Barby JS Loftis L Cummings L Meadow W Lemmon M Ubel P Should neonatologists give opinion withdrawing life-sustaining treatment. Pediatrics. (2016) 138(6):e20162585. 10.1542/peds.2016-2585 27940720

[33] Lantos J. Ethical problems in decision making in the neonatal ICU. N Engl J Med. (2018) 379(19):1851–60. 10.1056/NEJMra1801063 30403936

[34] Sankaran K Hedin E Hodgson-Viden H. Neonatal end of life care in a tertiary care centre in Canada: a brief report. Chin J Contemp Pediatr. (2016) 18(5):379–85. 10.7499/j.issn.1008-8830.2016.05.001 PMC7390367 27165583

[35] Carter B. End of life decisions for newborns: an ethical and compassionate process? Arch Dis Child Fetal Neonatal Ed. (2016) 2(10a):F92–3. 10.1136/archdischild-2015-309380 26542878

[36] Caeymaex L Jousselme C Vasilescu C Danan C Falissard B Bourrat M-M Perceived role in end-of-life decision making in the NICU affects long-term parental grief response. Arch Dis Child Fetal Neonatal Ed. (2013) 98(1):f26–31. 10.1136/archdischild-2011-301548 22732115

